# English Language Proficiency and Early School Attainment Among Children Learning English as an Additional Language

**DOI:** 10.1111/cdev.12615

**Published:** 2016-09-20

**Authors:** Katie E. Whiteside, Debbie Gooch, Courtenay F. Norbury

**Affiliations:** ^1^ Royal HollowayUniversity of London; ^2^ University College London

## Abstract

Children learning English as an additional language (EAL) often experience lower academic attainment than monolingual peers. In this study, teachers provided ratings of English language proficiency and social, emotional, and behavioral functioning for 782 children with EAL and 6,485 monolingual children in reception year (ages 4–5). Academic attainment was assessed in reception and Year 2 (ages 6–7). Relative to monolingual peers with comparable English language proficiency, children with EAL displayed fewer social, emotional, and behavioral difficulties in reception, were equally likely to meet curriculum targets in reception, and were more likely to meet targets in Year 2. Academic attainment and social, emotional, and behavioral functioning in children with EAL are associated with English language proficiency at school entry.

As a result of greater international mobility, an increasing proportion of children around the world are growing up learning multiple languages. For example, it has been estimated that 21.9% of young people, aged between 5 and 17 years, in the United States speak a language other than English in their home (U. S. Census Bureau, 2014). Additionally, 19.4% of children attending state‐funded primary schools in England speak English as an additional language (EAL; Department for Education, 2015). Such children are educated in English, however they have been exposed to a language other than English at home since infancy (Department for Education, 2015; Strand, Malmberg, & Hall, 2015). The proportion of children who speak EAL in England has been rising quite dramatically, from 8.7% in 2000, 11.6% in 2005, to 16% in 2010 (NALDIC, 2013). Because children are regarded as having EAL on the basis of language exposure in their home, the EAL label gives no indication of English language proficiency (Strand et al., 2015). Children with EAL are a heterogeneous group, with English language skills spanning the full continuum of proficiency (Strand et al., 2015). Bilingual speakers are frequently reported to display cognitive advantages, particularly in executive functioning, relative to monolingual speakers (Bialystok, Craik, Green, & Gollan, 2009). However, these advantages are not always realized in functional academic performance. For both children with EAL and their monolingual English‐speaking peers, English language proficiency may be a more prominent associate of academic and social, emotional, and behavioral profiles rather than EAL status.

In England, children with EAL, as a group, display poorer attainment throughout primary school than monolingual children. This trend is revealed in data from the 2014 national education assessments, which measured the attainment of all state‐funded primary school pupils who were at the end of their 1st year of school (reception year; ages 4–5), Year 2 (ages 6–7), and Year 6 (ages 10–11; Department for Education, 2014a, 2014b, 2014c). These assessments revealed that the attainment gap between children with EAL and monolingual peers is widest in the curriculum area of speaking in reception year, speaking and listening in Year 2, and reading in Year 6, though the attainment gap is not limited to language‐related subjects. Strand et al. (2015) analyzed national assessment data collected in 2013 and concluded that the attainment gap between children with EAL and monolingual peers narrows but is maintained, across primary school. Strand et al. also reported that the attainment gap is eliminated by Year 11 (ages 15–16), where students with EAL actually show better attainment in some areas of the curriculum relative to monolingual peers.

Strand et al. (2015) noted that there is considerable variation in academic attainment among children with EAL and sought to explore risk factors for low attainment. Male sex, younger relative age, low family and neighborhood socioeconomic status (SES), special education needs (SEN), and arriving in the United Kingdom part way through primary school were all associated with low academic attainment in Year 6 assessments in children with EAL. However, Strand et al. noted that English language proficiency is likely to be the most important predictor of attainment. A recent meta‐analysis reported moderate to strong positive associations between proficiency in the language of education and early literacy, reading, spelling, mathematics, and general academic attainment among bilingual children (Prevoo, Malda, Mesman, & van IJzendoorn, 2015). This is not surprising as proficiency in the language of education is required to understand the teacher, and language proficiency is a precursor for reading (Hoff, 2013; Prevoo et al., 2015).

Relatively little research has investigated how English language proficiency levels among children with EAL can influence the academic attainment gap between children with EAL and monolingual peers. In an analysis of attainment in Year 6 assessments, Strand and Demie (2005) reported that children with EAL who were fully fluent in English showed better attainment in all Year 6 assessment areas relative to monolingual children, though this difference was not significant after controlling for child characteristics including age, sex, SES, ethnicity, and SEN. In contrast, children with EAL who were not fully fluent in English performed poorer than monolingual children, even after controlling for child characteristics. Demie and Strand (2006) also found the same pattern of results when analyzing attainment by monolingual and EAL students in Year 11. These studies suggest that English language proficiency is an important factor in predicting how well children with EAL perform relative to monolingual peers in assessments at the end of primary and secondary school.

Whereas previous studies have focused on older children, Halle, Hair, Wandner, McNamara, and Chien (2012) found that English language proficiency also predicts how children with EAL perform relative to monolingual peers over the early school years. Specifically, when controlling for child, family, and school characteristics, Halle et al. found that children with EAL who were not proficient in English until first grade (ages 6–7), or later, showed lower reading and maths attainment in kindergarten (ages 5–6) than monolingual children. In contrast, children with EAL who were proficient in English at school entry showed comparable attainment in reading and math in kindergarten to monolingual children. These children also displayed greater growth in reading and math between kindergarten and eighth grade (ages 13–14), relative to monolingual children. This highlights potential academic advantages of having EAL, when coupled with good English language proficiency.

English language proficiency is also associated with social, emotional, and behavioral functioning in children with EAL. After controlling for child, family, and school characteristics, Halle et al. (2012) found that children with EAL who were proficient in English at school entry typically showed better behavior, attention, eagerness to learn, and organization between kindergarten (ages 5–6) and fifth‐grade (ages 10–11) than monolingual children. In contrast, children with EAL who were not proficient in English by first grade showed comparable behavior, but poorer attention, eagerness to learn, and organization between kindergarten and fifth grade, relative to monolingual children. Similarly, Winsler, Kim, and Richard (2014) found that Latino children with EAL and high English language proficiency showed greater social, emotional, and behavioral functioning at age 4 compared to monolingual English‐speaking children. In contrast, Latino children with EAL and low English language proficiency typically showed comparable social, emotional, and behavioral functioning to monolingual children. Other studies of primarily Latino children with EAL have similarly found that high English language proficiency is associated with greater social, emotional, and behavioral functioning (Dowdy, Dever, DiStefano, & Chin, 2011; Oades‐Sese, Esquivel, Kaliski, & Maniatis, 2011).

These findings are somewhat consistent with literature suggesting that bilingualism is associated with a range of cognitive advantages. For example, research has found that bilingual children display enhanced executive functioning relative to monolingual children, including enhanced inhibition (Calvo & Bialystok, 2014; Engel de Abreu, Cruz‐Santos, Tourinho, Martin, & Bialystok, 2012; Poarch & van Hell, 2012), working memory (Calvo & Bialystok, 2014), and task switching (Barac & Bialystok, 2012). However, many studies have not replicated the bilingual executive functioning advantage (Duñabeitia et al., 2014; Gathercole et al., 2014), and other studies have found that it is dependent on factors such as language use at home (Gathercole et al., 2010). Moreover, other research has suggested that enhanced executive functioning in bilingual children is dependent on having good proficiency in both languages (Engel de Abreu, Cruz‐Santos, & Puglisi, 2014). Thus, previous findings of enhanced academic attainment and social, emotional, and behavioral functioning in children with EAL, who demonstrate good English language proficiency, may reflect enhanced executive functioning in these children. Indeed, greater executive functioning is associated with greater academic attainment (St Clair‐Thompson & Gathercole, 2006; Stevenson, Bergwerff, Heiser, & Resing, 2014; Yeniad, Malda, Mesman, van IJzendoorn, & Pieper, 2013) and behavioral functioning (Ciairano, Visu‐Petra, & Settanni, 2007; Hughes & Ensor, 2011) in monolingual children.

Previous findings concerning the relation between English language proficiency and academic attainment and social, emotional, and behavioral functioning among children with EAL are difficult to interpret, as studies have not consistently considered the language proficiency of the monolingual comparison children. In order to make meaningful comparisons, children with EAL should be compared to monolingual children with comparable English language proficiency. This is because language proficiency among monolingual children is also associated with academic attainment and social, emotional, and behavioral functioning. For example, monolingual children with language impairment show poorer academic attainment (Dockrell, Ricketts, Palikara, Charman, & Lindsay, 2012; Tomblin, 2014) and greater social, emotional, and behavioral difficulties (Bretherton et al., 2014; McCabe, 2005; Yew & O'Kearney, 2013) relative to typically developing monolingual peers.

To our knowledge, only two studies have compared academic and social, emotional, and behavioral outcomes of children with EAL against monolingual peers with comparable language proficiency. One such study was carried out in Australia by Goldfeld, O'Connor, Mithen, Sayers, and Brinkman (2014). Goldfeld et al. analyzed population data from a teacher‐completed checklist, which measured development in the following areas in the 1st year of school: physical health and well‐being, social competence, emotional maturity, and language and cognition (including literacy, maths, and memory). Each child's English proficiency was determined on the basis of teacher ratings of their ability to use English (*very poor* or *poor *= not English proficient; *average, good,* or *very good *= English proficient). When controlling for demographic variables, English‐proficient children with EAL were equally likely to show vulnerable social competence, language, and cognition, and were less likely to show vulnerable emotional maturity and physical health and well‐being, compared to English‐proficient monolingual children. On the other hand, children with EAL who were not English proficient were more likely to show vulnerable development in all areas compared to English‐proficient monolingual children. However, monolingual children who were not English proficient were at the greatest risk of displaying vulnerable development in all areas. It is likely that the language difficulties experienced by the children with EAL and the monolingual children, who were deemed not English proficient, reflected different origins (Goldfeld et al., 2014), which may explain why these groups displayed different levels of developmental vulnerability. The language difficulties experienced by the monolingual children were perhaps more likely to reflect an underlying language impairment, whereas the English language difficulties experienced by the children with EAL may have reflected a lack of language exposure, an underlying language impairment, or both.

A similar study was recently carried out by McLeod, Harrison, Whiteford, and Walker (2016). McLeod et al. explored longitudinal academic and social, emotional, and behavioral outcomes of Australian children with EAL, and monolingual peers, whose parents reported that they either had concerns, or no concerns, about their child's speech and language at ages 4–5. At ages 4–5, 6–7, and 8–9, children with EAL showed comparable social, emotional, and behavioral functioning and academic attainment to monolingual peers with comparable speech and language concern, after controlling for demographic variables. Noticeably, in contrast to Goldfeld et al.'s (2014) findings, children with EAL did not show advantages in social, emotional, and behavioral functioning relative to monolingual peers with comparable speech and language concern. Children with EAL and speech and language concern typically did not differ significantly in academic attainment from both monolingual and EAL peers with no speech and language concern. In contrast, monolingual children with speech and language concern typically showed significantly poorer academic attainment relative to both monolingual and EAL peers with no speech and language concern. Thus, comparable to Goldfeld et al.'s (2014) findings, monolingual children with speech and language concern were at the greatest risk of low academic attainment.

## Current Study

In sum, research suggests that academic attainment and social, emotional, and behavioral functioning in children with EAL is dependent on English language proficiency. However, there is a need for more research to compare the academic and social, emotional, and behavioral profiles of children with EAL against monolingual peers with comparable English language proficiency. Additionally, previous research has reduced language proficiency to a binary variable (Goldfeld et al., 2014; McLeod et al., 2016) or used parent‐reported speech and language concern as a proxy for language proficiency (McLeod et al., 2016). The current study builds on previous research by using a continuous, psychometrically strong, measure of English language proficiency. This is advantageous as it allows children with EAL and monolingual peers to be compared across the continuum of language proficiency rather than just at low and typical levels of language proficiency.

The current study reports data from a UK‐based longitudinal population study of language development. The aim of this study was to compare children with EAL to monolingual peers, with comparable English language proficiency in the 1st year of school (reception year; ages 4–5); on social, emotional, and behavioral functioning in reception year; and on academic attainment in reception year and Year 2 (ages 6–7). This study has strong ecological validity as data from national assessments were analyzed to measure academic attainment. In order to investigate the functional impact of EAL status and English language proficiency levels, children were compared against curriculum targets that are used in the classroom. On the basis of previous findings, it was predicted that lower English language proficiency in reception year, among both children with EAL and monolingual peers, would be associated with greater social, emotional, and behavioral difficulties, and a lower likelihood of meeting curriculum targets both concurrently and in Year 2. Additionally, on the basis of previous findings, children with EAL were predicted to show comparable or fewer social, emotional, and behavioral difficulties in reception year relative to monolingual peers with comparable English language proficiency. In terms of academic attainment, children with EAL were predicted to be equally likely to meet curriculum targets in reception year, but more likely to meet and exceed curriculum targets in Year 2, relative to monolingual peers with comparable English language proficiency. Finally, children with EAL were predicted to be more likely to show progress in meeting curriculum targets between reception year and Year 2, relative to monolingual peers with comparable English language proficiency.

## Method

### Participants

This study reports data collected for 7,267 reception year children during the population survey phase of the Surrey Communication and Language in Education Study. Additionally, this study incorporates data from national curriculum assessments, provided by Surrey County Council, which were completed by the same children 2 years later. All children who started reception year in a state‐maintained school in Surrey, England, in September 2011 were eligible to take part in the study (*N *=* *12,398). Of the 263 eligible schools who were invited to participate, 161 schools participated (61% of all eligible schools). Between May and July 2012, teachers completed an online questionnaire for 7,267 children (59% of all eligible children) who were in the last term of reception year. The research team covered the costs of supply teaching for a day to allow teachers time to complete the questionnaire for each child in their class who was taking part in the study. As data were anonymous to the research team and direct assessment of individual children was not required, an opt‐out consent procedure was adopted. Parents received an information sheet via schools and had the opportunity to opt out of allowing anonymized teacher ratings of their child's academic attainment, language, and behavior to be submitted to the study. Twenty families opted out at this stage. The study protocol was developed in collaboration with Surrey County Council education officials and was granted ethical approval by the Ethics Committee at Royal Holloway, University of London.

Of the final sample of 7,267 children, 6,485 (89%) children were monolingual English speaking and 782 (11%) children spoke EAL. Children were regarded as speaking EAL if teachers reported that the main language spoken in the child's home was not English. The 2015 School Census found that 19.4% of children in state‐funded primary schools in England spoke EAL and 12% of children in state‐funded primary schools in Surrey spoke EAL (Department for Education, 2015). Therefore, the proportion of children with EAL in this sample is somewhat lower than the national proportion but comparable to the proportion in Surrey.

Over 64 different languages were represented in the sample of children with EAL. The most frequently reported first language was Urdu (*n *=* *83, 11% of EAL sample), followed by Polish (*n *=* *76, 10%), Portuguese (*n *=* *47, 6%), Bengali (*n *=* *43, 5%), and Panjabi (*n *=* *40, 5%). The first language was unknown for 44 (6%) children. The top languages reported in this sample are consistent with the 2012 School Census, which revealed that Urdu, Polish, Panjabi, Bengali, and Portuguese were, respectively, the most frequently reported first languages other than English for children in state‐funded schools in Surrey (NALDIC, 2012a). The top languages spoken in this sample are also comparable to the most frequently reported first languages, other than English, for children in state‐funded schools in England: Urdu, Panjabi, Bengali, Polish, and Somali (NALDIC, 2012a).

The children with EAL were from 122 state‐maintained schools across Surrey and the monolingual children were from 161 state‐maintained schools across Surrey. The EAL sample consisted of 402 (51%) boys and 380 (49%) girls and the monolingual sample consisted of 3,312 (51%) boys and 3,173 (49%) girls. All children were aged between 4 years 9 months (57 months) and 5 years 10 months (70 months) when teachers completed the questionnaires. As shown in Table 1, the children with EAL and monolingual children did not significantly differ in age. Income Deprivation Affecting Children Index (IDACI; McLennan et al., 2011) rank scores were obtained using the children's home postcodes to provide a measure of neighborhood deprivation. England has been divided up into small geographical areas, and all areas have been ranked according to the proportion of children resident in each area who live in families deemed to be income deprived due to being in receipt of certain means tested benefits (McLennan et al., 2011). IDACI rank scores can range from 1 to 32,482, with lower scores assigned to areas with proportionally more children living in income‐deprived families. IDACI rank scores for the EAL sample ranged from 1,730 to 32,459, and IDACI rank scores for the monolingual sample ranged from 731 to 32,474. As shown in Table 1, the monolingual children had significantly higher IDACI rank scores and thus were from less deprived neighborhoods than the children with EAL.

**Table 1:** Descriptive Statistics for Continuous Variables for Monolingual Children and Children With EAL

Variable	Monolingual		EAL		*U*	*p*	*r*
	*M* (*SD*)	*Mdn* (*IQR*)	*M* (*SD*)	*Mdn* (*IQR*)			
Age in months	64.16 (3.55)	64.00 (6.00)	64.20 (3.51)	64.00 (6.00)	2,516,452.00	.728	< .01
IDACI rank score^a^	21,963.52 (7,670.95)	22,748.00 (12,768.00)	18,512.54 (8,439.69)	18,384.50 (14,928.75)	1,937,300.00	< .001	−.13
CCC–S score^b^	8.64 (8.64)	7.00 (12.00)	15.13 (10.51)	14.00 (15.00)	1,573,021.00	< .001	−.20
SDQ total difficulties^c^	5.42 (5.20)	4.00 (6.00)	6.01 (5.29)	5.00 (7.00)	2,342,472.00	< .001	−.04

Note.

EAL = English as an additional language; IDACI = Income Deprivation Affecting Children Index; CCC–S = Children's Communication Checklist–Short; SDQ = Strengths and Difficulties Questionnaire; *Mdn* = median; *IQR* = interquartile range.

^a^Greater IDACI rank scores indicate lower neighborhood deprivation. ^b^Greater CCC–S scores indicate lower English language proficiency. ^c^Greater SDQ total difficulties scores indicate greater social, emotional, and behavioral difficulties.

### Measures and Procedures

The teacher questionnaire was completed when the children were at the end of reception year (ages 4–5) and consisted of a short version of the Children's Communication Checklist–2 (CCC–2; Bishop, 2003), the Strengths and Difficulties Questionnaire (SDQ; Goodman, 1997), and the Early Years Foundation Stage Profile (EYFSP; Standards and Testing Agency, 2012). Additionally, Surrey County Council provided data from national curriculum assessments, which were completed when the children were in Year 2 (ages 6–7).

#### Children's Communication Checklist–Short

The CCC–S is a short version of the CCC–2 (Bishop, 2003), which is a well‐validated language screening measure that can discriminate between children with language impairment and typically developing children (Norbury, Nash, Baird, & Bishop, 2004). The CCC–S contains items that best discriminated children with language impairment from typically developing peers in Norbury et al.'s (2004) validation study. The CCC–S has good internal consistency and excellent agreement with the full CCC–2 (Norbury et al., 2015). The respondent first provides a range of background information about the child, including sex, date of birth, home postcode, and first language. The next part of the CCC–S contains six items describing communicative errors and seven items describing communicative strengths (e.g., “you can have an enjoyable, interesting conversation with him/her”). The respondent rates how often the child displays each communicative error or strength using a 4‐point scale: *rarely or never (less than once a week), occasionally (once a week), regularly (once or twice a day)*, or *frequently or always (several times a day)*. The six items regarding communicative errors were scored from 0 (*rarely or never*) to 3 (*frequently or always*), whereas the seven items regarding communicative strengths were reverse scored (3 = *rarely or never*, 0 = *frequently or always*). All 13 items were summed to create a total CCC–S score (maximum = 39), with high scores reflecting lower English language proficiency.

#### Strengths and Difficulties Questionnaire

The SDQ is a screening measure of social, emotional, and behavioral functioning developed for use with 4‐ to 16‐year‐olds (Goodman, 1997). A review of 48 studies concluded that the SDQ has strong psychometric properties, including satisfactory reliability, good construct validity, and a good capacity to identify children who have a disorder (Stone, Otten, Engels, Vermulst, & Janssens, 2010). The SDQ is made up of 25 items, with five items for each of the five subscales: emotional symptoms, conduct problems, hyperactivity, peer problems, and prosocial behavior. The respondent rates the extent each item applies to the child on a 3‐point scale (*not true, somewhat true*, or *certainly true;* scored from 0 to 2). Scores on the first four subscales were summed to provide a total difficulties score (maximum = 40), with high scores reflecting greater social, emotional, and behavioral difficulties.

#### Early Years Foundation Stage Profile

The EYFSP is a measure of attainment completed by teachers during the last term of reception year for children attending state‐maintained schools in England (Standards and Testing Agency, 2012). Using a 3‐point scale (*emerging, expected,* or *exceeding*), teachers rate the extent to which each child has met the expected level of development across 17 early learning goals. Children were regarded as achieving a “good level of development” if they achieved at least the expected level of development across 12 key early learning goals (Department for Education, 2014a). These 12 goals relate to the following areas of learning: communication and language; physical development; personal, social, and emotional development; literacy; and mathematics.

#### Year 2 Assessments

Children attending state‐maintained schools in England complete national curriculum assessments, known as Key Stage 1 assessments, in Year 2 (ages 6–7; Department for Education, 2014c). Teachers determine each child's level of attainment in the following five subjects: mathematics, science, reading, writing, and speaking and listening. Because the expected level of attainment is Level 2 (Department for Education, 2014c), for the purposes of this study, children were regarded as performing on target if they achieved Level 2 or above in all five subjects and were regarded as performing below target if they achieved Level 1 or below in one or more subject. Children were regarded as performing above target if they achieved Level 3 or above in three or more subjects and Level 2 in any remaining subjects.

### Missing Data

Home postcodes were unavailable for 148 monolingual children and 26 children with EAL and were replaced with the postcode for the child's school. SDQ and EYFSP data were missing for one child, and EYFSP data were missing for a further six children. Year 2 assessment results were missing for 870 (12%) children. Missing SDQ, EYFSP, and Year 2 assessment data were not imputed: Children with missing data were simply excluded from relevant analyses. A greater proportion of children with EAL (*n *=* *134, 17%) had missing Year 2 assessment results relative to monolingual children, *n *=* *736, 11%; χ^2^(1) = 22.17, *p *<* *.001, φ = .06. A Mann–Whitney *U* test revealed that CCC–S scores did not significantly differ between children whose Year 2 assessment results were missing (median [*Mdn*] = 7.00; interquartile range [*IQR*] = 12) and children whose results were available (Mdn = 7.00, IQR =12.00; *U *=* *2,758, 380.00, *Z *= −0.42, *p *=* *.674, *r *<* *.01), which indicates that these groups did not differ in English language proficiency. Additionally, IDACI rank scores did not significantly differ between children whose Year 2 assessment results were missing (Mdn = 22,358.00, IQR = 13,401.25) and children whose results were available (Mdn = 22,378.00, IQR = 13,229.00; *U *=* *2,730,023.50, *Z *=* *−0.91, *p *=* *.364, *r *=* *.01), which indicates that these groups also did not differ in neighborhood deprivation.

### Data Analysis

First, Mann–Whitney *U* tests were run to explore whether children with EAL and monolingual children differed on CCC–S scores (English language proficiency) and SDQ total difficulties scores. Chi‐square tests were then run to explore whether children with EAL and monolingual children differed in their likelihood to achieve the following academic attainment outcomes, before language proficiency was considered: perform at a good level of development in reception year, perform on target in Year 2 assessments, perform above target in Year 2 assessments, and progress from a performing below a good level of development in reception year to performing on target in Year 2. The latter analysis only used data from children who performed below a good level of development in reception year and explored whether children with EAL were more likely to show progress in meeting curriculum targets, between reception year and Year 2 than monolingual peers. Following this, hierarchical binary logistic regression and hierarchical multiple regression were used to explore how children with EAL compared to monolingual peers on each binary academic attainment outcome, and on SDQ total difficulties scores, after first controlling for language proficiency (unadjusted model) and then after additionally controlling for demographic variables (adjusted model).

EAL status, CCC–S scores, and the CCC–S × EAL Status interaction term were entered into the first, unadjusted, model of each regression. Within each unadjusted model, regression coefficients and odds ratios for EAL status reveal how children with EAL compare to monolingual peers on each attainment outcome when CCC–S scores are 0 (i.e., when English language proficiency is high). Likewise, regression coefficients and odds ratios for CCC–S scores reveal the association between CCC–S scores and each attainment outcome when EAL status is 0 (i.e., statistics for monolingual children). The CCC–S × EAL Status interaction term reveals whether the association between CCC–S scores and each attainment outcome differs for children with EAL relative to monolingual peers. In other words, the interaction term reveals whether the association between EAL status and each attainment outcome differs across the continuum of CCC–S scores. Sex, age in months, and IDACI rank scores (neighborhood deprivation) were additionally entered into the second, adjusted, model of each regression to examine whether the associations revealed in the unadjusted model held after these variables, which are known to be associated with behavioral functioning and academic attainment, were held constant.

## Results

**Figure 1:**
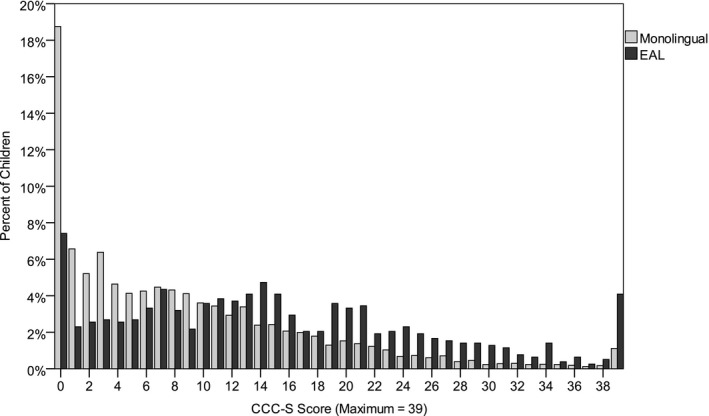
The percentage of monolingual children and children with English as an additional language (EAL) who received each score on the Children's Communication Checklist–Short (CCC–S). Greater CCC–S scores indicate lower English language proficiency.

Figure 1 displays the distribution of scores on the CCC–S for monolingual children and children with EAL. Most monolingual children received low CCC–S scores, indicating high teacher‐rated English language proficiency, and fewer children are represented as CCC–S scores increase. In contrast, the distribution of scores for children with EAL is more evenly spread across the entire range. As shown in Table 1, children with EAL, as a group, had significantly higher CCC–S scores and thus lower English language proficiency than monolingual children. Children with EAL also had significantly higher SDQ total difficulties scores than monolingual children (see Table 1), which implies that they had greater social, emotional, and behavioral difficulties. Additionally, as shown in Table 2, children with EAL were significantly less likely than monolingual children to achieve a good level of development in reception year and perform on target, or above target, in Year 2 assessments. However, all effects were small. Furthermore, children with EAL and monolingual children were equally likely to progress from a performing below a good level of development in reception year to performing on target in Year 2 (see Table 2).

**Table 2:** The Percentage of Monolingual Children and Children With EAL Who Achieved Each Attainment Outcome

Attainment outcome	Monolingual %	EAL %	χ^2^(*df*)	*p*	φ
GLD in reception	59	45	54.46 (1)	< .001	.09
On target in Year 2	86	82	5.72 (1)	.017	.03
Above target in Year 2	31	23	18.83 (1)	< .001	.05
Below GLD in reception but on target in Year 2	69	70	0.06 (1)	.806	< .01

Note.

EAL = English as an additional language; GLD = good level of development.

**Figure 2:**
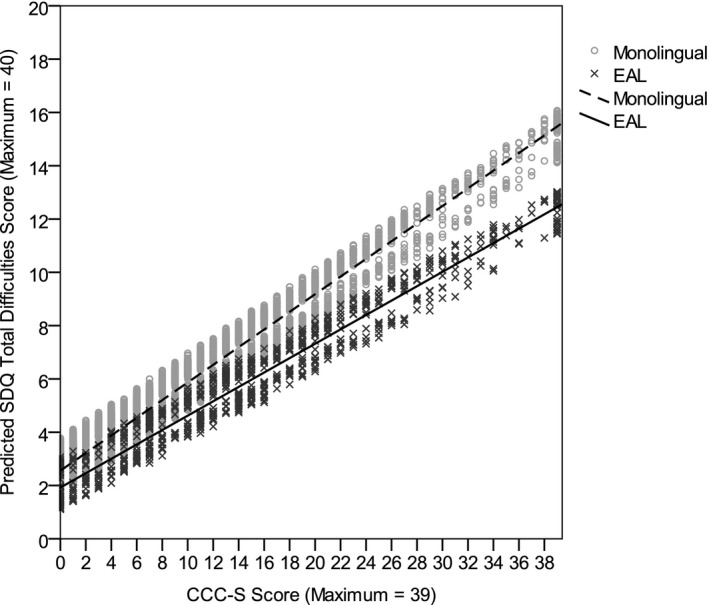
Predicted Strengths and Difficulties Questionnaire (SDQ) total difficulties scores by Children's Communication Checklist–Short (CCC–S) scores for monolingual children and children with English as an additional language (EAL) after controlling for demographic variables. Greater CCC–S scores indicate lower English language proficiency and greater SDQ total difficulties scores indicate greater social, emotional, and behavioral difficulties.

Hierarchical multiple regression examined the association between EAL status and total difficulties scores on the SDQ, after controlling for language proficiency in the unadjusted model and additionally controlling for demographic variables in the adjusted model. The unadjusted model significantly predicted total difficulties scores, *F*(3, 7,262) = 1,047.84, *p *<* *.001, and explained 30% of the variance. As shown in Table 3, higher CCC–S scores (i.e., lower English language proficiency) significantly predicted greater total difficulties scores and EAL status significantly predicted lower total difficulties scores. Moreover, there was a significant CCC–S × EAL Status interaction; compared to monolingual children, an increase in CCC–S scores among children with EAL was associated with a smaller increase in total difficulties scores (see Figure 2). These results imply that children with EAL experience fewer social, emotional, and behavioral difficulties than monolingual peers with comparable English language proficiency, and this EAL advantage is greater among children with lower English language proficiency. Controlling for demographic variables in the adjusted model did not change the associations revealed in the unadjusted model (see Table 3), though prediction was significantly improved, *F*(3, 7,259) = 47.54, *p *<* *.001, and a further 1% of the variance was explained. In total, the adjusted model explained 32% of the variance and significantly predicted total difficulties scores, *F*(6, 7,259) = 557.76, *p *<* *.001.

**Table 3:** Hierarchical Multiple Regression Predicting Total Difficulties Scores on the SDQ in Reception Year (*n* = 7,266)

Variable	*b*	*SE*	β	*t*	*p*
Unadjusted model					
EAL	−0.63	.28	−.04	−2.23	.026
CCC–S score	0.33	.01	.58	52.91	< .001
CCC–S × EAL	−0.06	.02	−.07	−3.81	< .001
Constant	2.56	.08		33.41	< .001
Adjusted model					
EAL	−0.63	.28	−.04	−2.23	.026
CCC–S score	0.32	.01	.55	48.89	< .001
CCC–S × EAL	−0.06	.02	−.07	−3.78	< .001
Male sex	1.16	.10	.11	11.29	< .001
Age in months	−0.03	.01	−.02	−2.22	.027
IDACI rank	< −0.01	< .01	−.04	−3.67	< .001
Constant	4.71	.96		4.90	< .001

Note.

SDQ = Strengths and Difficulties Questionnaire; EAL = English as an additional language; CCC–S = Children's Communication Checklist–Short; IDACI = Income Deprivation Affecting Children Index.

Hierarchical logistic regression was then run to predict which children achieved a good level of development in reception year. The unadjusted model was significant, χ^2^(3) = 2,799.63, *p *<* *.001, and explained between 32% (Cox–Snell *R* ^2^) and 43% (Nagelkerke *R* ^2^) of the variance. As shown in Table 4, higher CCC–S scores, reflecting lower English language proficiency, were associated with significantly lower odds of achieving a good level of development. EAL status was not a significant predictor of good level of development status and there was no significant CCC–S × EAL Status interaction. This implies that, across the continuum of English language proficiency, children with EAL and monolingual peers with comparable language proficiency were equally likely to achieve a good level of development in reception year. Controlling for demographic variables in the adjusted model did not change the associations revealed in the unadjusted model (see Table 4), though prediction was significantly improved, χ^2^(3) = 153.86, *p *<* *.001. The adjusted model was significant, χ^2^(6) = 2,953.49, *p *<* *.001, and explained between 33% (Cox–Snell *R* ^2^) and 45% (Nagelkerke *R* ^2^) of the variance.

**Table 4:** Hierarchical Logistic Regression Predicting Which Children Achieved a Good Level of Development on the EYFSP in Reception Year (*n* = 7,260)

Variable	*b*	*SE*	Wald	*p*	Odds ratio (95% CI)
Unadjusted model					
EAL	0.25	.20	1.59	.207	1.28 (0.87, 1.89)
CCC–S score	−0.20	.01	1,420.33	< .001	0.82 (0.81, 0.83)
CCC–S × EAL	0.02	.01	1.28	.258	1.02 (0.99, 1.04)
Constant	2.01	.05	1,508.96	< .001	
Adjusted model					
EAL	0.24	.20	1.48	.223	1.28 (0.86, 1.89)
CCC–S score	−0.19	.01	1,256.44	< .001	0.83 (0.82, 0.84)
CCC–S × EAL	0.01	.01	0.77	.381	1.01 (0.98, 1.04)
Male sex	−0.56	.06	89.58	< .001	0.57 (0.51, 0.64)
Age in months	0.07	.01	68.00	< .001	1.07 (1.06, 1.09)
IDACI rank	< 0.01	< .01	4.84	.028	1.00 (1.00, 1.00)
Constant	−2.46	.56	19.45	< .001	

Note.

EYFSP = Early Years Foundation Stage Profile; EAL = English as an additional language; CCC–S = Children's Communication Checklist–Short; IDACI = Income Deprivation Affecting Children Index.

The next analyses focused on academic attainment 2 years later. First, hierarchical logistic regression was run to predict on target performance in Year 2 assessments. The unadjusted model was significant, χ^2^(3) = 1,265.86, *p *<* *.001, and explained between 18% (Cox–Snell *R* ^2^) and 32% (Nagelkerke *R* ^2^) of the variance. As shown in Table 5, higher CCC–S scores, reflecting lower English language proficiency in reception year, were associated with significantly lower odds of performing on target in Year 2. There was no significant CCC–S × EAL Status interaction; however, EAL status was associated with significantly higher odds of performing on target in Year 2. This indicates that children with EAL were more likely to meet academic targets in Year 2 relative to monolingual peers with comparable English language proficiency in reception year. When demographic variables were controlled in the adjusted model, this EAL advantage remained (see Table 5) and prediction was significantly improved, χ^2^(3) = 110.89, *p *<* *.001. The adjusted model was significant, χ^2^(6) = 1,376.75, *p *<* *.001, and explained between 19% (Cox–Snell *R* ^2^) and 34% (Nagelkerke *R* ^2^) of the variance.

**Table 5:** Hierarchical Logistic Regression Predicting on Target Performance in Year 2 Assessments (*n* = 6,397)

Variable	*b*	*SE*	Wald	*p*	Odds ratio (95% CI)
Unadjusted model					
EAL	0.64	.32	4.02	.045	1.90 (1.01, 3.56)
CCC–S score	−0.14	< .01	826.48	< .001	0.87 (0.86, 0.88)
CCC–S × EAL	0.01	.01	0.32	.570	1.01 (0.98, 1.03)
Constant	3.49	.08	1,788.76	< .001	
Adjusted model					
EAL	0.81	.32	6.26	.012	2.25 (1.19, 4.26)
CCC–S score	−0.13	.01	711.69	< .001	0.87 (0.87, 0.88)
CCC–S × EAL	0.01	.01	0.18	.670	1.01 (0.98, 1.03)
Male sex	−0.21	.09	6.23	.013	0.81 (0.68, 0.96)
Age in months	0.04	.01	10.87	.001	1.04 (1.02, 1.07)
IDACI rank	< 0.01	< .01	93.95	< .001	1.00 (1.00, 1.00)
Constant	−0.06	.79	0.01	.934	

Note.

EAL = English as an additional language; CCC–S = Children's Communication Checklist–Short; IDACI = Income Deprivation Affecting Children Index.

**Figure 3:**
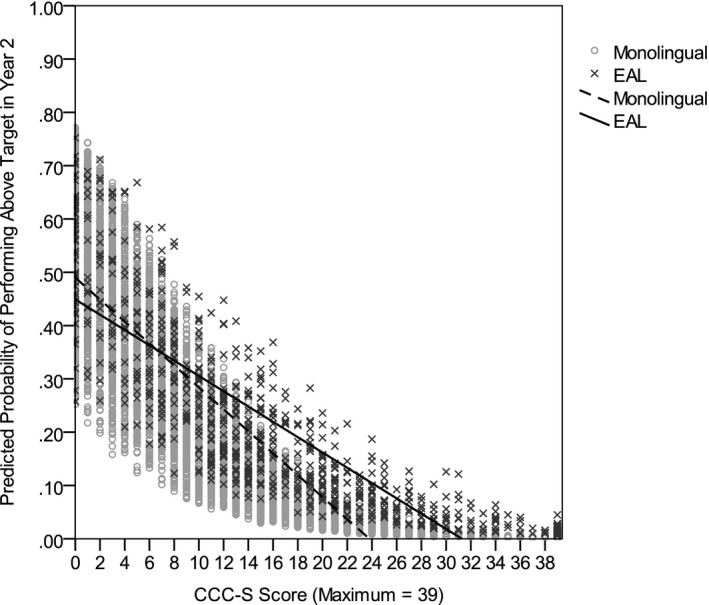
Predicted probability of performing above target in Year 2 assessments by Children's Communication Checklist–Short (CCC–S) scores for monolingual children and children with English as an additional language (EAL) after controlling for demographic variables. Greater CCC–S scores indicate lower English language proficiency.

The next hierarchical logistic regression predicted above target performance in Year 2 assessments. The unadjusted model was significant, χ^2^(3) = 1,266.80, *p *<* *.001, and explained between 18% (Cox–Snell *R* ^2^) and 25% (Nagelkerke *R* ^2^) of the variance. As shown in Table 6, higher CCC–S scores, reflecting lower English language proficiency in reception year, were associated with significantly lower odds of performing above target in Year 2. EAL status did not significantly predict above target performance. Thus, when CCC–S scores were 0, which indicates high English language proficiency, children with EAL and monolingual peers were equally likely to exceed Year 2 targets. However, there was a significant CCC–S × EAL Status interaction; as CCC–S scores increased, reflecting lower English language proficiency in reception year, children with EAL were more likely to perform above target in Year 2 assessments relative to monolingual peers with equivalent CCC–S scores (see Figure 3). Controlling for demographic variables in the adjusted model did not change these associations (see Table 6), though prediction was significantly improved, χ^2^(3) = 248.39, *p *<* *.001. The adjusted model was significant, χ^2^(6) = 1,515.19, *p *<* *.001, and explained between 21% (Cox–Snell *R* ^2^) and 30% (Nagelkerke *R* ^2^) of the variance.

**Table 6:** Hierarchical Logistic Regression Predicting Above Target Performance in Year 2 Assessments (*n* = 6,397)

Variable	*b*	*SE*	Wald	*p*	Odds ratio (95% CI)
Unadjusted model					
EAL	−0.14	.17	0.70	.404	0.87 (0.62, 1.21)
CCC–S score	−0.16	.01	718.37	< .001	0.85 (0.84, 0.86)
CCC–S × EAL	0.05	.01	13.97	< .001	1.05 (1.03, 1.08)
Constant	0.27	.04	38.11	< .001	
Adjusted model					
EAL	−0.01	.17	< 0.01	.965	0.99 (0.71, 1.39)
CCC–S score	−0.15	.01	613.39	< .001	0.86 (0.85, 0.87)
CCC–S × EAL	0.05	.01	13.35	< .001	1.05 (1.02, 1.08)
Male sex	0.09	.06	2.17	.140	1.10 (0.97, 1.24)
Age in months	0.08	.01	87.19	< .001	1.09 (1.07, 1.10)
IDACI rank	< 0.01	< .01	150.76	< .001	1.00 (1.00, 1.00)
Constant	−6.31	.59	115.61	< .001	

Note.

EAL = English as an additional language; CCC–S = Children's Communication Checklist–Short; IDACI = Income Deprivation Affecting Children Index.

The final hierarchical logistic regression predicted progression from performing below a good level of development in reception year to performing on target in Year 2. The unadjusted model was significant, χ^2^(3) = 442.95, *p *<* *.001, and explained between 15% (Cox–Snell *R* ^2^) and 21% (Nagelkerke *R* ^2^) of the variance. As shown in Table 7, higher CCC–S scores, reflecting lower English language proficiency in reception year, were associated with significantly lower odds of performing on target in Year 2. There was no significant CCC–S × EAL Status interaction; however, EAL status was associated with significantly higher odds of performing on target in Year 2. This indicates that children with EAL, who were academically underachieving in reception year, were more likely to go on and meet academic targets in Year 2 relative to monolingual peers with comparable language proficiency and academic attainment in reception year. When demographic variables were controlled in the adjusted model, this EAL advantage remained (see Table 7) and prediction was significantly improved, χ^2^(3) = 60.14, *p *<* *.001. The adjusted model was significant, χ^2^(6) = 503.09, *p *<* *.001, and explained between 17% (Cox–Snell *R* ^2^) and 24% (Nagelkerke *R* ^2^) of the variance.

**Table 7:** Hierarchical Logistic Regression Predicting Progression From Performing Below a Good Level of Development in Reception Year to Performing on Target in Year 2 (*n* = 2,723)

Variable	*b*	*SE*	Wald	*p*	Odds ratio (95% CI)
Unadjusted model					
EAL	0.91	.39	5.32	.021	2.48 (1.15, 5.36)
CCC–S score	−0.10	.01	306.32	< .001	0.91 (0.90, 0.92)
CCC–S × EAL	−0.01	.02	0.25	.618	0.99 (0.96, 1.02)
Constant	2.38	.11	504.56	< .001	
Adjusted model					
EAL	1.08	.40	7.33	.007	2.94 (1.35, 6.42)
CCC–S score	−0.10	.01	277.66	< .001	0.91 (0.90, 0.92)
CCC–S × EAL	−0.01	.02	0.34	.557	0.99 (0.96, 1.02)
Male sex	−0.09	.10	0.89	.346	0.91 (0.76, 1.10)
Age in months	0.02	.01	2.08	.150	1.02 (0.99, 1.05)
IDACI rank	< 0.01	< .01	57.09	< .001	1.00 (1.00, 1.00)
Constant	0.24	.88	0.07	.789	

Note.

EAL = English as an additional language; CCC–S = Children's Communication Checklist–Short; IDACI = Income Deprivation Affecting Children Index.

## Discussion

This study explored associations between teacher‐rated English language proficiency in the 1st year of school (reception year) and concurrent social, emotional, and behavioral functioning and academic attainment, as well as academic attainment 2 years later, in children with EAL and monolingual peers. As predicted, lower English language proficiency, in both children with EAL and monolingual peers, was associated with greater social, emotional, and behavioral difficulties in reception year and a lower likelihood of meeting curriculum targets in reception year and meeting or exceeding curriculum targets in Year 2. Lower English language proficiency, in both groups, was also associated with a lower likelihood of progressing from performing below target in reception year to performing on target in Year 2. Thus, low levels of English language proficiency at school entry represent a key risk factor for social, emotional, and behavioral difficulties, and persistent academic difficulties among both children with EAL and their monolingual peers.

Before English language proficiency was considered, children with EAL showed greater social, emotional, and behavioral difficulties than monolingual children and were less likely to achieve curriculum targets in reception year and achieve, or exceed, curriculum targets in Year 2. Nevertheless, children with EAL and monolingual children were equally likely to progress from performing below target in reception year to performing on target in Year 2. However, results were different when language proficiency was considered. Relative to monolingual peers with comparable English language proficiency, children with EAL displayed fewer social, emotional, and behavioral difficulties in reception year. Moreover, this EAL behavioral advantage became greater as English language proficiency decreased. Additionally, consistent with expectations, children with EAL were equally likely to meet curriculum targets in reception year and were more likely to meet curriculum targets in Year 2, relative to monolingual peers with comparable levels of English language proficiency in reception. Although children with EAL and monolingual peers with high English language proficiency were equally likely to exceed Year 2 targets, children with EAL became more likely to exceed Year 2 targets than monolingual peers as English language proficiency decreased. Finally, children with EAL were more likely to progress from a performing below target in reception to performing on target in Year 2, relative to monolingual peers with comparable English language proficiency in reception. These associations all held both before and after demographic variables were taken into account.

As noted in a previous study by Strand and Demie (2005), the current study highlights that caution is needed when interpreting data from national assessments for children with EAL as a group. Data from the national assessments in England indicate that children with EAL show poorer attainment throughout primary school compared to monolingual children (Strand et al., 2015). However, results from the current study, as well as from previous research (Demie & Strand, 2006; Goldfeld et al., 2014; Halle et al., 2012; Prevoo et al., 2015; Strand & Demie, 2005), suggest that academic attainment among children with EAL is dependent on English language proficiency. Indeed, the current study found that children with EAL show comparable, or better, academic attainment relative to monolingual peers with comparable English language proficiency. As noted by Strand et al. (2015), children with EAL are a heterogeneous group, with English language skills spanning the full continuum of proficiency. Findings from the current study support Strand and Demie's (2005) and Strand et al.'s (2015) recommendation that in order to determine the required support for individual children with EAL, it is important to consider their English language proficiency rather than just their EAL status.

This study is consistent with research reporting that greater English language proficiency in children with EAL is associated with greater academic attainment (Demie & Strand, 2006; Goldfeld et al., 2014; Halle et al., 2012; Prevoo et al., 2015; Strand & Demie, 2005) and greater social, emotional, and behavioral functioning (Dowdy et al., 2011; Goldfeld et al., 2014; Halle et al., 2012; Oades‐Sese et al., 2011; Winsler et al., 2014). However, there are some inconsistencies between this study and previous research concerning how children with EAL compare to monolingual peers on academic and social, emotional, and behavioral outcomes. These inconsistencies likely reflect methodological differences in the way English language proficiency was determined and the use of different measures of social, emotional, and behavioral functioning and academic attainment. Additionally, few previous studies have considered the language proficiency of the monolingual comparison children (e.g., Halle et al., 2012; Strand & Demie, 2005; Winsler et al., 2014), though Goldfeld et al. (2014) and McLeod et al. (2016) are notable exceptions. Nevertheless, results from this study are consistent with previous findings that children with EAL, who have good English language proficiency, show comparable academic attainment (Goldfeld et al., 2014; Halle et al., 2012; McLeod et al., 2016) and fewer social, emotional, and behavioral difficulties (Goldfeld et al., 2014; Halle et al., 2012; Winsler et al., 2014) at school entry, relative to monolingual peers, and show greater academic progress over the early school years (Halle et al., 2012).

Bilingual children are often reported to have cognitive advantages, in particular enhanced executive functioning, compared to monolingual children (Barac & Bialystok, 2012; Bialystok et al., 2009; Calvo & Bialystok, 2014; Engel de Abreu et al., 2012; Poarch & van Hell, 2012). Additionally, research has suggested that enhanced executive functioning in bilingual children is dependent on having good proficiency in both languages (Engel de Abreu et al., 2014). Greater executive functioning is also associated with greater academic attainment (St Clair‐Thompson & Gathercole, 2006; Stevenson et al., 2014; Yeniad et al., 2013) and behavioral functioning (Ciairano et al., 2007; Hughes & Ensor, 2011) generally, leading to the expectation that children with EAL, particularly those with good English language proficiency, would show behavioral and academic advantages relative to monolingual peers. The results from the current study gave a more mixed picture. Although children with EAL demonstrated no advantages in meeting curriculum targets in reception year, children with EAL did demonstrate advantages in social, emotional, and behavioral functioning in reception year; in meeting curriculum targets in Year 2; and in showing progress in meeting targets between reception year and Year 2. However, these advantages only appeared when children with EAL were compared against monolingual peers with comparable English language proficiency in reception year. Moreover, academic advantages for most children with EAL were limited to meeting curriculum targets. Only children with EAL and low English language proficiency displayed advantages in exceeding curriculum targets in Year 2, relative to monolingual peers with comparable language proficiency in reception year. As executive functioning was not measured in this study, it is uncertain whether these advantages in academic attainment and social, emotional, and behavioral functioning reflected enhanced executive functioning among children with EAL. Indeed, these advantages may reflect other factors, such as cultural or home environment differences. The relation between EAL status, English language proficiency, executive functioning, and academic and behavioral outcomes are a fruitful avenue for future research.

In this study, discrepancies between children with EAL and monolingual peers in social, emotional, and behavioral functioning in reception year and in academic attainment in Year 2 became greater as language proficiency decreased. This may indicate that bilingualism may be a protective factor against some of the difficulties associated with low language proficiency or language impairment (Engel de Abreu et al., 2014). However, these findings may also reflect the different or multifaceted origins of the language difficulties in these two groups. For many children with EAL, low English language proficiency in reception year reflects a lack of language exposure, whereas it may be more indicative of an underlying language impairment in monolingual children. Indeed, although all children should have received nearly a full academic year of exposure to English by the time teachers rated their language proficiency, exposure to English prior to school entry is likely to have been variable among the children with EAL, with some children experiencing little to no exposure to English prior to school entry (NALDIC, 2012b). Given the assessment methods, the nature of the population sample and the number of different languages represented within the population, it was not possible to screen for language proficiency in the child's first or home language. Future studies should quantify both exposure to English prior to school entry and level of language proficiency in the home language in order to better understand unexplained variance in the academic attainment and social, emotional, and behavioral functioning of children with EAL.

As language impairment is associated with social, emotional, and behavioral difficulties (Bretherton et al., 2014; Yew & O'Kearney, 2013) and poor academic attainment (Dockrell et al., 2012) in monolingual children, future research should further consider how to distinguish language impairment from limited language exposure in children with EAL in order to identify those who will likely overcome their initial English language difficulties and to target support more effectively for those children who may struggle to catch up. Indeed, identifying language impairment in children learning EAL is a key challenge faced by practitioners (De Lamo White & Jin, 2011; Hasson, Camilleri, Jones, Smith, & Dodd, 2013). Although this is a growing area of research, there is still a lack of appropriate measures to identify language impairment in bilingual children, particularly in children from diverse first language backgrounds (Kohnert, 2010; Paradis, 2010; Paradis, Schneider, & Duncan, 2013). A further challenge for practitioners is in determining how best to intervene. Although it is important to support development of both languages and encourage families to continue to provide rich interactions and experiences in their first language, for clinicians and educators it may not be practical to offer direct instruction in other languages. This is particularly true in the United Kingdom where over 300 different languages are represented by pupils in primary and secondary schools (NALDIC, 2012a). The findings of the current study suggest that increasing proficiency in English, or the main language of instruction, during the early school years or prior to school entry will improve social, emotional, and behavioral functioning and academic performance. The impact of such interventions should be evaluated using randomized controlled trials.

A strength of this study is that it used a population cohort of children, who were all in the same school year and had been exposed to academic English for the same amount of time. Additionally, unlike most previous studies on the association between English language proficiency, academic attainment, and social, emotional, and behavioral functioning in children with EAL, this study considered the language proficiency of the monolingual comparison children. Through the use of national assessments, it was also possible to compare children against attainment targets used in the classroom and thus delineate the functional impact of English language proficiency levels and EAL status. Another strength of this study reflects the use of standard checklists of language and social, emotional, and behavioral functioning, which have strong psychometric properties (Norbury et al., 2015; Stone et al., 2010). Nevertheless, this study is limited through the use of indirect measures of language and social, emotional, and behavioral functioning and a lack of multiple informants. Although the brief language screen used was necessary to allow such a large sample size, directly assessing each child with a battery of language tests may have provided a better indication of each child's English language proficiency and would have decreased the reliance on teacher ratings. Indeed, the same teacher provided ratings of language, academic attainment, and social, emotional, and behavioral functioning in reception year for each child, which may have inflated associations between these measures. Nevertheless, teacher ratings of English language proficiency in reception year were predictive of both academic attainment in reception year as well as independently reported levels of academic attainment in Year 2. A further potential issue concerns the fact that 39 of the 166 participating schools only contributed data from monolingual children. It is possible that variance in the school environment may have contributed to some of the findings. However, after excluding all children from these 39 schools, all effects remained the same in each regression model (see Appendix S1).

### Conclusion

English language proficiency in children with EAL at school entry is predictive of concurrent academic attainment and social, emotional, and behavioral functioning, as well as academic attainment 2 years later. These findings highlight that children with EAL are a heterogeneous group, and caution is required when interpreting data from national assessments for children with EAL, without considering English language proficiency. Although previous research has highlighted cognitive advantages associated with bilingualism, in this study, children with EAL displayed no advantage in academic attainment in reception year. However, children with EAL displayed advantages in social, emotional, and behavioral functioning in reception year and a functional advantage in meeting curriculum targets in Year 2, relative to monolingual peers with comparable levels of English language proficiency. Future research should explore whether these advantages are related to enhanced executive functioning in children with EAL. Future research should also explore how to distinguish children with EAL at school entry who are likely to have persistent language deficits, from those with more transient difficulties associated with limited exposure to English, in order to provide more targeted support. Findings from this study suggest that a focus on boosting English language proficiency in the early school years, or prior to school entry, among children with EAL will improve social, emotional, and behavioral profiles and attenuate the existing academic attainment gap between children with EAL and monolingual peers.

## Supporting information


**Appendix S1.** Analysis After Excluding Children From Schools Which Only Contributed Data From Monolingual Children
