# Supplementary material for: English Language Proficiency and Early School Attainment Among Children Learning English as an Additional Language
Source: Child Dev. 2016 Sep 20;88(3):812–27. doi: 10.1111/cdev.12615 (PMC5434859; doi:10.1111/cdev.12615)
Supplement: Supplementary file 1 — Appendix S1. Analysis After Excluding Children From Schools Which Only Contributed Data From Monolingual Children [file CDEV-88-812-s001.docx]

**Supporting Information for Online Publication Only**

**Appendix S1: Analysis After Excluding Children From Schools Which Only Contributed Data From Monolingual Children**

This section presents the results of the whole analysis after excluding 1,252 monolingual children who attended one of the 39 schools which only contributed data from monolingual children. All children within this sample (*N* = 6,015) attended one of 122 state-maintained schools across Surrey. The participants in this sample were 5,233 (87%) English-speaking monolingual children and 782 (13%) children with English as an additional language (EAL). The EAL sample consisted of 402 males (51%) and 380 females (49%) and the monolingual sample consisted of 2,673 males (51%) and 2,560 females (49%). All children were aged between 4 years 9 months (57 months) and 5 years 10 months (70 months) when reception year data were collected. As shown in Table S1, the children with EAL and monolingual children did not significantly differ in age. Table S1 also shows that the monolingual children had significantly higher Income Deprivation Affecting Children Index (IDACI) rank scores, and thus were from less deprived neighbourhoods, than the children with EAL.

Strengths and Difficulties Questionnaire (SDQ) and Early Years Foundation Stage Profile (EYFSP) data were missing for one child and EYFSP data were missing for a further four children. Year 2 assessment results were missing for 708 (12%) children. A greater proportion of children with EAL (*n* = 134, 17%) had missing Year 2 assessment results relative to monolingual children (*n* = 574, 11%; χ^2^(1) = 24.91, *p* < .001, Phi *= .06*). Children with missing SDQ, EYFSP or Year 2 assessment data were excluded from relevant analyses. The data analysis procedure outlined in the main manuscript was followed in this analysis.

**Results**

Figure S1 displays the distribution of scores on the CCC-S for monolingual children and children with EAL. Most monolingual children received low CCC-S scores, indicating high teacher-rated English language proficiency, and fewer children are represented as CCC-S scores increase. In contrast, the distribution of scores for children with EAL is more evenly spread across the entire range. As shown in Table S1, children with EAL, as a group, had significantly higher CCC-S scores, and thus lower teacher-rated English language proficiency, than monolingual children. Children with EAL also had significantly higher SDQ total difficulties scores than monolingual children (see Table S1), which implies that they had greater social, emotional, and behavioural difficulties. Additionally, as shown in Table S2, children with EAL were significantly less likely than monolingual children to achieve a good level of development in reception year and perform above target in Year 2 assessments. However, these effects were small. Furthermore, children with EAL and monolingual children were equally likely to perform on target in Year 2 assessments and progress from a performing below a good level of development in reception year to performing on target in Year 2 (see Table S2).


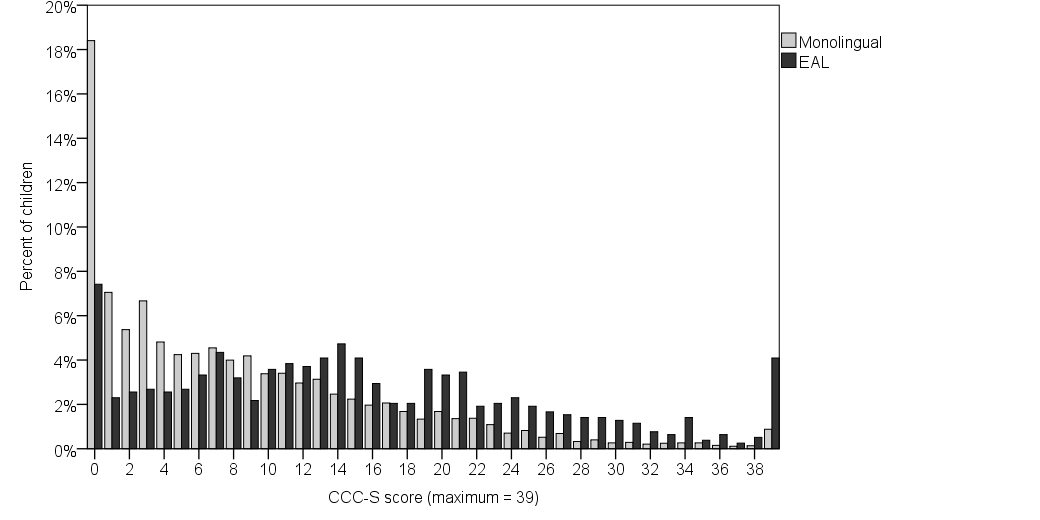


Figure S1. The percentage of monolingual children and children with EAL who received each score on the CCC-S.

Table S1

*Descriptive Statistics for Continuous Variables for Monolingual Children and Children with EAL*

| Variable | Monolingual | | EAL | |  |  |  |
| --- | --- | --- | --- | --- | --- | --- | --- |
|  | *M* (*SD*) | *Mdn* (*IQR*) | *M* (*SD)* | *Mdn* (*IQR*) | *U* | *p* | *r* |
| Age in months | 64.18 (3.56) | 64.00 (6.00) | 64.20 (3.51) | 64.00 (6.00) | 2,039,375.50 | .882 | <.01 |
| IDACI rank score^a^ | 21,540.08 (7,784.84) | 22,316.00 (12,916.00) | 18,512.54 (8,439.69) | 18,384.50 (14,928.75) | 1,622,045.50 | < .001 | -.12 |
| CCC-S score^b^ | 8.50 (8.51) | 6.00 (12.00) | 15.13 (10.51) | 14.00 (15.00) | 1,255,472.50 | < .001 | -.23 |
| SDQ total difficulties^c^ | 5.46 (5.20) | 4.00 (6.00) | 6.01 (5.29) | 5.00 (7.00) | 1,902,675.50 | .002 | -.04 |

^a^Greater IDACI rank scores indicate lower neighbourhood deprivation. ^b^Greater CCC-S scores indicate lower English language proficiency. ^c^Greater SDQ total difficulties scores indicate greater social, emotional, and behavioural difficulties.

Table S2

*The Percentage of Monolingual Children and Children with EAL who Achieved Each Attainment Outcome*

| Attainment outcome | Monolingual | EAL | χ^2^*(df)* | *p* | Phi |
| --- | --- | --- | --- | --- | --- |
| GLD in reception | 59% | 45% | 56.93 (1) | < .001 | .10 |
| On target in Year 2 | 85% | 82% | 3.14 (1) | .077 | .02 |
| Above target in Year 2 | 30% | 23% | 13.14 (1) | < .001 | .05 |
| Below GLD in reception but on target in Year 2 | 68% | 70% | 0.75 (1) | .388 | .02 |

*Note.* GLD = good level of development.

Hierarchical multiple regression was run to examine the association between EAL status and total difficulties scores on the SDQ, after controlling for language proficiency in the unadjusted model and additionally controlling for demographic variables in the adjusted model. The unadjusted model significantly predicted total difficulties scores, *F*(3, 6010) = 893.59, *p* < .001, and explained 31% of the variance. As shown in Table S3, higher CCC-S scores (i.e. lower English language proficiency) significantly predicted greater total difficulties scores and EAL status significantly predicted lower total difficulties scores. Moreover, there was a significant CCC-S by EAL status interaction; compared to monolingual children, an increase in CCC-S scores among children with EAL was associated with a smaller increase in total difficulties scores (see Figure S2). These results imply that children with EAL experience fewer social, emotional, and behavioural difficulties than monolingual peers with comparable English language proficiency and this EAL advantage is greater among children with lower English language proficiency. Controlling for demographic variables in the adjusted model did not change the associations revealed in the unadjusted model (see Table S3), though prediction was significantly improved, *F*(3, 6007) = 39.00, *p* < .001, and a further 1% of the variance was explained. In total, the adjusted model explained 32% of the variance and significantly predicted total difficulties scores, *F*(6, 6007) = 474.77, *p* < .001.

Table S3

*Hierarchical Multiple Regression Predicting Total Difficulties Scores on the SDQ in Reception Year (N = 6014)*

| Variable | *b* | *SE* | β | *t* | *p* |
| --- | --- | --- | --- | --- | --- |
| Unadjusted model |  |  |  |  |  |
| EAL | -0.64 | 0.28 | -.04 | -2.24 | .025 |
| CCC-S score | 0.34 | 0.01 | .59 | 48.33 | < .001 |
| CCC-S x EAL | -0.07 | 0.02 | -.09 | -4.35 | < .001 |
| Constant | 2.56 | 0.08 |  | 30.19 | < .001 |
| Adjusted model |  |  |  |  |  |
| EAL | -0.63 | 0.28 | -.04 | -2.21 | .027 |
| CCC-S score | 0.33 | 0.01 | .57 | 45.09 | < .001 |
| CCC-S x EAL | -0.07 | 0.02 | -.09 | -4.39 | < .001 |
| Male sex | 1.15 | 0.11 | .11 | 10.22 | < .001 |
| Age in months | -0.02 | 0.02 | -.02 | -1.42 | .156 |
| IDACI rank score | < -0.01 | < 0.01 | -.04 | -3.68 | < .001 |
| Constant | 4.11 | 1.05 |  | 3.91 | < .001 |


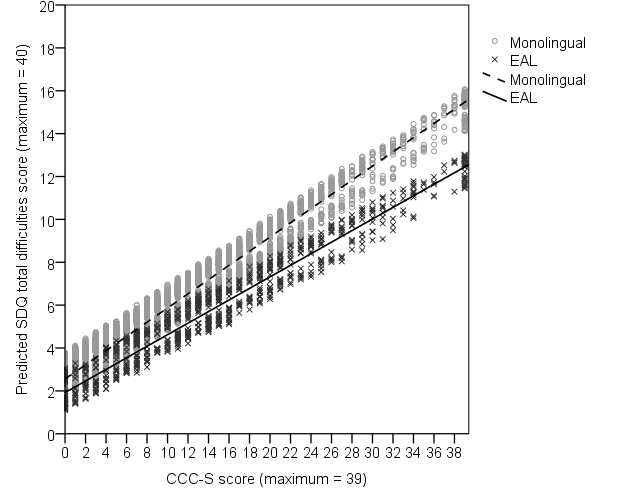


Figure S2. Predicted SDQ total difficulties scores by CCC-S scores for monolingual children and children with EAL, after controlling for demographic variables. Greater CCC-S scores indicate lower English language proficiency and greater SDQ total difficulties scores indicate greater social, emotional, and behavioural difficulties.

Hierarchical logistic regression was then run to examine the association between EAL status and achieving a good level of development in reception year, after controlling for language proficiency in the unadjusted model and additionally controlling for demographic variables in the adjusted model. The unadjusted model was significant, χ^2^(3) = 2,359.58, *p* < .001, and explained between 32% (Cox & Snell *R^2^*) and 44% (Nagelkerke *R^2^*) of the variance. As shown in Table S4, higher CCC-S scores, reflecting lower English language proficiency, were associated with significantly lower odds of achieving a good level of development. EAL status was not a significant predictor of good level of development status and there was no significant CCC-S by EAL status interaction. This implies that, across the continuum of English language proficiency, children with EAL and monolingual children with comparable language proficiency were equally likely to achieve a good level of development in reception year. Controlling for demographic variables in the adjusted model did not change these associations (see Table S4), though prediction was significantly improved, χ^2^(3) = 105.64, *p* < .001. The adjusted model was significant, χ^2^(6) = 2,465.22, *p* < .001, and explained between 34% (Cox & Snell *R^2^*) and 45% (Nagelkerke *R^2^*) of the variance.

Table S4

*Hierarchical Logistic Regression Predicting Which Children Achieved a Good Level of Development on the EYFSP in Reception Year (N = 6010)*

| Variable | *b* | *SE* | Wald | *p* | Odds ratio (95% *CI*) |
| --- | --- | --- | --- | --- | --- |
| Unadjusted model |  |  |  |  |  |
| EAL | 0.22 | 0.20 | 1.20 | .274 | 1.24 (0.84, 1.83) |
| CCC-S score | -0.20 | 0.01 | 1163.55 | < .001 | 0.82 (0.81, 0.83) |
| CCC-S x EAL | 0.02 | 0.01 | 1.74 | .187 | 1.02 (0.99, 1.05) |
| Constant | 2.04 | 0.06 | 1248.61 | < .001 |  |
| Adjusted model |  |  |  |  |  |
| EAL | 0.22 | 0.20 | 1.15 | .284 | 1.24 (0.84, 1.85) |
| CCC-S score | -0.19 | 0.01 | 1045.08 | < .001 | 0.83 (0.82, 0.84) |
| CCC-S x EAL | 0.02 | 0.01 | 1.31 | .253 | 1.02 (0.99, 1.05) |
| Male sex | -0.55 | 0.07 | 69.24 | < .001 | 0.58 (0.51, 0.66) |
| Age in months | 0.06 | 0.01 | 34.96 | < .001 | 1.06 (1.04, 1.08) |
| IDACI rank score | < 0.01 | < .01 | 7.73 | .005 | 1.00 (1.00, 1.00) |
| Constant | -1.54 | 0.61 | 6.25 | .012 |  |

The next analyses focused on academic attainment two years later. Firstly, hierarchical logistic regression was run to predict on target performance in Year 2 assessments. The unadjusted model was significant, χ^2^(3) = 1,057.27, *p* < .001, and explained between 18% (Cox & Snell *R^2^*) and 31% (Nagelkerke *R^2^*) of the variance. As shown in Table S5, higher CCC-S scores, reflecting lower English language proficiency in reception year, were associated with significantly lower odds of performing on target in Year 2. There was no significant CCC-S by EAL status interaction, however EAL status was associated with significantly higher odds of performing on target in Year 2. This shows that children with EAL were more likely to meet academic targets in Year 2 than monolingual peers with comparable language proficiency in reception year. When demographic variables were controlled in the adjusted model, this EAL advantage remained (see Table S5) and prediction was significantly improved, χ^2^(3) = 93.25, *p* < .001. The adjusted model was significant, χ^2^(6) = 1,150.52, *p* < .001, and explained between 19% (Cox & Snell *R^2^*) and 34% (Nagelkerke *R^2^*) of the variance.

Table S5

*Hierarchical Logistic Regression Predicting On Target Performance in Year 2 Assessments (N = 5307)*

| Variable | *b* | *SE* | Wald | *p* | Odds ratio (95% *CI*) |
| --- | --- | --- | --- | --- | --- |
| Unadjusted model |  |  |  |  |  |
| EAL | 0.76 | 0.32 | 5.58 | .018 | 2.14 (1.14, 4.02) |
| CCC-S score | -0.14 | 0.01 | 675.01 | < .001 | 0.87 (0.86, 0.88) |
| CCC-S x EAL | 0.01 | 0.01 | 0.27 | .606 | 1.01 (0.98, 1.03) |
| Constant | 3.37 | 0.09 | 1456.67 | < .001 |  |
| Adjusted model |  |  |  |  |  |
| EAL | 0.90 | 0.33 | 7.65 | .006 | 2.46 (1.30, 4.67) |
| CCC-S score | -0.13 | 0.01 | 576.50 | < .001 | 0.88 (0.87, 0.89) |
| CCC-S x EAL | < 0.01 | 0.01 | 0.13 | .723 | 1.00 (0.98, 1.03) |
| Male sex | -0.24 | 0.09 | 6.85 | .009 | 0.79 (0.66, 0.94) |
| Age in months | 0.04 | 0.01 | 10.74 | .001 | 1.04 (1.02, 1.07) |
| IDACI rank score | < 0.01 | < 0.01 | 76.44 | < .001 | 1.00 (1.00, 1.00) |
| Constant | -0.27 | 0.84 | 0.10 | .747 |  |

The next hierarchical logistic regression predicted above target performance in Year 2 assessments. The unadjusted model was significant, χ^2^(3) = 1,064.96, *p* < .001, and explained between 18% (Cox & Snell *R^2^*) and 26% (Nagelkerke *R^2^*) of the variance. As shown in Table S6, higher CCC-S scores, reflecting lower English language proficiency in reception year, were associated with significantly lower odds of performing above target in Year 2. EAL status did not significantly predict above target performance. Thus, when CCC-S scores were 0, which reflects high English language proficiency, children with EAL and monolingual peers were equally likely to exceed Year 2 targets. However, there was a significant CCC-S by EAL status interaction; as CCC-S scores increased, reflecting lower English language proficiency in reception year, children with EAL were more likely to perform above target in Year 2 relative to monolingual peers with equivalent CCC-S scores (see Figure S3). Controlling for demographic variables in the adjusted model did not change these associations (see Table S6), though prediction was significantly improved, χ^2^(3) = 194.72, *p* < .001. The adjusted model was significant, χ^2^(6) = 1,259.68, *p* < .001, and explained between 21% (Cox & Snell *R^2^*) and 30% (Nagelkerke *R^2^*) of the variance.

Table S6

*Hierarchical Logistic Regression Predicting Above Target Performance in Year 2 Assessments (N = 5307)*

| Variable | *b* | *SE* | Wald | *p* | Odds ratio (95% *CI*) |
| --- | --- | --- | --- | --- | --- |
| Unadjusted model |  |  |  |  |  |
| EAL | -0.09 | 0.17 | 0.30 | .584 | 0.91 (0.65, 1.27) |
| CCC-S score | -0.17 | 0.01 | 578.85 | < .001 | 0.84 (0.83, 0.85) |
| CCC-S x EAL | 0.06 | 0.01 | 18.29 | < .001 | 1.07 (1.03, 1.10) |
| Constant | 0.22 | 0.05 | 20.71 | < .001 |  |
| Adjusted model |  |  |  |  |  |
| EAL | 0.04 | 0.18 | 0.04 | .835 | 1.04 (0.74, 1.46) |
| CCC-S score | -0.16 | 0.01 | 496.99 | < .001 | 0.85 (0.84, 0.86) |
| CCC-S x EAL | 0.06 | 0.01 | 16.90 | < .001 | 1.06 (1.03, 1.10) |
| Male sex | 0.09 | 0.07 | 1.56 | .211 | 1.09 (0.95, 1.25) |
| Age in months | 0.08 | 0.01 | 60.46 | < .001 | 1.08 (1.06, 1.10) |
| IDACI rank score | < 0.01 | < 0.01 | 127.94 | < .001 | 1.00 (1.00, 1.00) |
| Constant | -5.97 | 0.65 | 83.33 | < .001 |  |


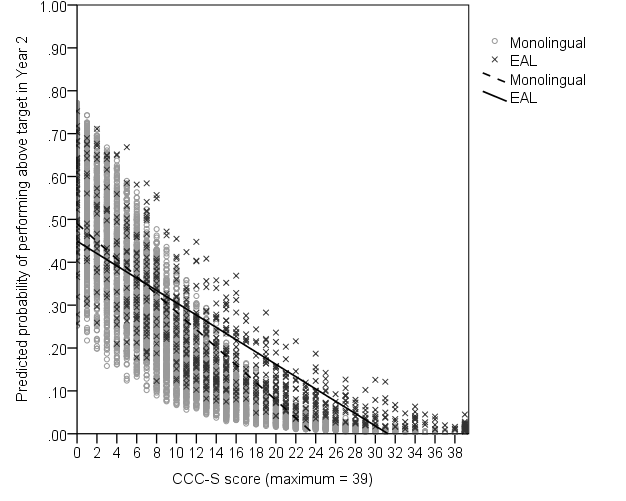


Figure S3. Predicted probability of performing above target in Year 2 assessments by CCC-S scores for monolingual children and children with EAL, after controlling for demographic variables. Greater CCC-S scores indicate lower English language proficiency.

The final hierarchical logistic regression predicted progression from performing below a good level of development in reception year to performing on target in Year 2. The unadjusted model was significant, χ^2^(3) = 352.35, *p* < .001, and explained between 14% (Cox & Snell *R^2^*) and 20% (Nagelkerke *R^2^*) of the variance. As shown in Table S7, higher CCC-S scores, reflecting lower English language proficiency in reception year, were associated with significantly lower odds of performing on target in Year 2. There was no significant CCC-S by EAL status interaction, however EAL status was associated with significantly higher odds of performing on target in Year 2. This indicates that children with EAL, who were academically underachieving in reception year, were more likely to go on and meet academic targets in Year 2 relative to monolingual peers with comparable language proficiency and academic attainment in reception year. When demographic variables were controlled in the adjusted model, this EAL advantage remained (see Table S7) and prediction was significantly improved, χ^2^(3) = 47.25, *p* < .001. The adjusted model was significant, χ^2^(6) = 399.60, *p* < .001, and explained between 16% (Cox & Snell *R^2^*) and 23% (Nagelkerke *R^2^*) of the variance.

Table S7

*Hierarchical Logistic Regression Predicting Progression From Performing Below a Good Level of Development in Reception Year to Performing On Target in Year 2 (N = 2257)*

| Variable | *b* | *SE* | Wald | *p* | Odds ratio (95% *CI*) |
| --- | --- | --- | --- | --- | --- |
| Unadjusted model |  |  |  |  |  |
| EAL | 1.06 | 0.40 | 7.14 | .008 | 2.88 (1.33, 6.26) |
| CCC-S score | -0.10 | 0.01 | 234.33 | < .001 | 0.91 (0.90, 0.92) |
| CCC-S x EAL | -0.01 | 0.02 | 0.47 | .495 | 0.99 (0.96, 1.02) |
| Constant | 2.23 | 0.12 | 376.23 | < .001 |  |
| Adjusted model |  |  |  |  |  |
| EAL | 1.19 | 0.40 | 8.84 | .003 | 3.29 (1.50, 7.20) |
| CCC-S score | -0.09 | 0.01 | 207.96 | < .001 | 0.91 (0.90, 0.92) |
| CCC-S x EAL | -0.01 | 0.02 | 0.60 | .439 | 0.99 (0.96, 1.02) |
| Male sex | -0.13 | 0.10 | 1.63 | .202 | 0.88 (0.72, 1.07) |
| Age in months | 0.03 | 0.01 | 3.31 | .069 | 1.03 (1.00, 1.06) |
| IDACI rank score | < 0.01 | < 0.01 | 42.87 | < .001 | 1.00 (1.00, 1.00) |
| Constant | -0.26 | 0.95 | 0.07 | .788 |  |
